# Molecular Mechanisms of Autism Spectrum Disorder

**DOI:** 10.3390/cells15080688

**Published:** 2026-04-14

**Authors:** Tanya Gandhi, Charles C. Lee

**Affiliations:** Department of Comparative Biomedical Sciences, School of Veterinary Medicine, Louisiana State University, Baton Rouge, LA 70803, USA

## 1. Introduction to the Special Issue

Autism spectrum disorder (ASD) is a heterogeneous neurodevelopmental condition characterized by differences in social communication and restricted, repetitive patterns of behavior. With global prevalence estimates exceeding 1% and substantially higher rates reported in some surveillance cohorts, ASD represents a major public health issue [1]. Decades of research have identified diverse molecular etiologies contributing to ASD; however, further work is required to refine these insights, clarify their convergence and developmental trajectories, and enhance their translational relevance [2].

The causes of ASD encompass several individually rare genetic conditions and idiopathic cases that converge on common neurobiological pathways [1]. Advances in genomics, epigenetics, stem-cell modeling, and system-level analyses have illuminated multiple interconnected molecular mechanisms underlying ASD, including synaptic and network dysregulation, epigenetic alterations, immune response and mitochondria dysfunction, calcium signaling abnormalities, and altered neurodevelopmental timing. The contributions in this Special Issue of *Cells*, “Molecular Mechanisms of Autism Spectrum Disorder,” deepen our understanding of the molecular mechanisms underlying ASD and shared neurobiological pathways.

For instance, ASD is characterized by marked genetic heterogeneity. Individual risk genes typically account for a small proportion of cases, yet many converge on shared key neurobiological processes, encompassing neuronal structure, function, and connectivity. In this issue, Belenska-Todorova et al. present the results of their whole-exome sequencing study, which further supports this mechanistic convergence [3]. Variants in genes involved in synaptic signaling, gene expression, cell cycle regulation, mitochondrial function, organelle trafficking, ciliogenesis, spectrin structure, and neuronal homeostasis highlight how disruptions in multiple aspects of neural development and function intersect in ASD pathophysiology. Notably, novel single-nucleotide variants were identified in the SPATA5, CEP120, BBS5, SETD1A, TRAK1, VPS13B, and DDX3X genes, implicated in many of the above functions during neurodevelopment. The identification of these novel variants advances the understanding of molecular mechanisms of autism and expands the diagnostic framework, with potential relevance for therapeutic strategies.

Increasingly, ASD is also conceptualized as a disorder of neurodevelopmental timing and epigenetic programming. The review by Ayoub on critical developmental periods highlights fetal and early postnatal windows during which folate availability, oxidative stress, inflammation, and microglial immune function may influence long-term neurodevelopmental outcomes [4]. Folate-dependent methylation is essential for DNA regulation and transcriptional control, and insufficient folate availability during these critical periods may alter gene expression patterns associated with ASD risk. Further, inadequate microglial immune responses during fetal development may promote oxidative stress and inflammatory changes in the developing brain, disrupting typical neurodevelopmental processes.

In a unique maternal obesity study, Allan et al. further refine this concept by distinguishing pre-conception from gestational effects using IVF and embryo transfer paradigms [5]. Pre-conception high-fat diet exposure alone was sufficient to induce ASD-like behaviors in male offspring. Cortical transcriptomic analysis revealed dysregulation and isoform shifts in ASD-associated genes, including Homer1, while the whole-genome bisulfite sequencing of hippocampal tissue demonstrated hypomethylation of an alternative Homer1 promoter associated with increased expression of the short isoform Homer1a, known to disrupt synaptic scaffolding and signaling. These findings suggest that ASD-related risk may be programmed prior to conception, involve isoform-specific epigenetic modifications, and may exhibit sex-specific vulnerability.

Neuroinflammation and oxidative stress are increasingly implicated in subsets of ASD. The induced pluripotent stem cell (iPSC)-derived astrocyte and neuron study by Mostafavi Abdolmaleky et al. showed increased expression of inflammatory mediators, including TGFB1, TGFB2, and IL6, alongside DNA methylation alterations that parallel findings in postmortem ASD brain samples [6]. Morphological and cellular changes were also observed, including increased astrocyte size with reduced growth rate, as well as reduced neuronal arborization, spine size, growth rate, and migration. These results suggest altered neuron–glial interactions and support the use of patient-derived iPSC models to recapitulate molecular and cellular alterations associated with ASD.

Similarly, the Rett syndrome review by Gonçalez et al. describes immune dysregulation in RTT associated with the abnormal activity of macrophages, microglia, lymphocytes, and non-immune cells, resulting in altered inflammatory mediator release and changes in the NF-κB signaling pathway [7]. The review also highlights mitochondrial dysfunction, including impaired energy production, altered calcium storage, and disrupted redox balance, which may contribute to oxidative stress and neuronal dysfunction in RTT.

Altered calcium signaling represents another recurring molecular feature in ASD. AlShawaf et al. demonstrate stage-specific dysregulation during neural differentiation using an iPSC-based calcium signaling approach [8]. ASD-derived iPSCs displayed elevated ATP-evoked calcium responses, whereas differentiated ASD neurons exhibited reduced ATP-evoked calcium responses but increased calcium responses to KCl and (S)-3,5-dihydroxyphenylglycine (DHPG) stimulation compared to controls. These findings suggest abnormalities in calcium signaling during neurodevelopmental stages. Given the central role of calcium in transcriptional regulation, synaptic plasticity, and mitochondrial function, such dysregulation may contribute to aberrant developmental trajectories in ASD.

At the systems level, circuit dysregulation contributes to the behavioral phenotype of ASD. Restricted repetitive behaviors (RRBs) are among the core diagnostic features of ASD, yet the underlying circuit mechanisms are not well understood. In this issue, Farmer et al. demonstrate altered functional connectivity in C58 mice in their somatosensory, striatal, thalamic, and sensory processing networks; this study links sensory processing abnormalities with repetitive behaviors [9]. Environmental enrichment attenuated RRBs by altering connectivity in somatosensory, pain, and visual networks. These findings suggest that aberrant sensory processing pathways may contribute to the development of repetitive behaviors in ASD.

Animal models have provided significant mechanistic insight into ASD-related gene function, circuit alterations, and behavioral phenotypes. However, they are limited in their ability to model the developmental timeline of the human brain and human-specific transcriptional paradigms. The review by Ranjan and Bhattacharya on functional models of ASD highlights the increasing use of patient-derived iPSCs, 3D organoids, and assembloids [10]. These systems can reproduce gene expression changes, epigenetic signatures, calcium signaling abnormalities, and neuronal morphological phenotypes relevant to ASD, making them useful for translational research and developing therapeutic interventions.

## 2. Conclusions

Together, the articles in this Special Issue signify the convergence of diverse genetic and environmental factors on interconnected molecular and cellular processes affecting neurodevelopmental processes and circuitry, resulting in heterogeneous ASD symptomatology. However, integrating and validating findings across diverse experimental model systems remains a major challenge for the field. Further, additional human studies are required to explore epigenetic and immune alterations, circuit and neuronal dysfunction, and behavioral outcomes across developmental timepoints. Additionally, translational approaches directed at environmental modulation and sensory processing require further development. Moreover, elucidating gender-dependent neurodevelopmental trajectories is crucial to understanding gender-specific findings and the prevalence of ASD in experimental models.

Collectively, this Special Issue provides insight into the molecular, cellular, and circuit-level mechanisms underlying ASD pathophysiology and highlights the importance of neurodevelopmental model systems for advancing mechanistic understanding and the development of future targeted interventions.

## Data Availability

No new data were created or analyzed for this article. Data sharing is not applicable.
